# Genetic susceptibility for neurodevelopmental disorders in children born to women with epilepsy

**DOI:** 10.1093/braincomms/fcag078

**Published:** 2026-03-16

**Authors:** Mathilde Kinge-Rasmussen, Emilie Willoch Olstad, Marte-Helene Bjørk, Hedvig Marie Egeland Nordeng, Manuela M X Tan, Kristina Gervin, Kaja Kristine Selmer

**Affiliations:** Department of Research and Innovation, Division of Clinical Neuroscience, Oslo University Hospital, 0424 Oslo, Norway; Institute of Clinical Medicine, University of Oslo, 0318 Oslo, Norway; Department of Research and Innovation, Division of Clinical Neuroscience, Oslo University Hospital, 0424 Oslo, Norway; Pharmacoepidemiology and Drug Safety Research Group, Department of Pharmacy, Faculty of Mathematics and Natural Sciences, University of Oslo, 0371 Oslo, Norway; UiO:RealArt Convergence Environment, University of Oslo, 0371 Oslo, Norway; Department of Clinical Medicine, University of Bergen, 5020 Bergen, Norway; Department of Neurology, Haukeland University Hospital, 5021 Bergen, Norway; Pharmacoepidemiology and Drug Safety Research Group, Department of Pharmacy, Faculty of Mathematics and Natural Sciences, University of Oslo, 0371 Oslo, Norway; UiO:RealArt Convergence Environment, University of Oslo, 0371 Oslo, Norway; Department of Child Health and Development, Norwegian Institute of Public Health, 0404 Oslo, Norway; Department of Neurology, Oslo University Hospital, 0450 Oslo, Norway; Department of Research and Innovation, Division of Clinical Neuroscience, Oslo University Hospital, 0424 Oslo, Norway; Pharmacoepidemiology and Drug Safety Research Group, Department of Pharmacy, Faculty of Mathematics and Natural Sciences, University of Oslo, 0371 Oslo, Norway; UiO:RealArt Convergence Environment, University of Oslo, 0371 Oslo, Norway; Department of Research and Innovation, Division of Clinical Neuroscience, Oslo University Hospital, 0424 Oslo, Norway; National Centre for Epilepsy, Full Member of EpiCARE European Reference Network for Rare and Complex Epilepsy, Oslo University Hospital, 0424 Oslo, Norway

**Keywords:** polygenic risk score, epilepsy, attention-deficit/hyperactivity disorder, autism spectrum disorder

## Abstract

Children of women with epilepsy have higher rates of neurodevelopmental disorders including attention-deficit/hyperactivity disorder (ADHD) and autism spectrum disorder (ASD), than the general population. This likely reflects a complex gene-environment interplay, but the specific contribution of genetic susceptibility, independent of *in utero* antiseizure medication exposure, remains unclear. The objective of this study was to evaluate whether polygenic risk scores (PRSs) for ADHD and ASD are elevated in these children compared to the general population and to assess associations with childhood neurodevelopmental traits. We analysed genetic and questionnaire data from the Norwegian Mother, Father and Child Cohort Study (MoBa). We included children born to women with epilepsy (*n* = 422) and children of mothers without epilepsy (*n* = 73 300). The parent-reported neurodevelopmental traits hyperactivity, inattention, social difficulties, language difficulties, repetitive behaviour and motor difficulties were measured using *z* scores at ages 0.5, 1.5, 3, 5 and 8 years. The genetic susceptibility was calculated as PRSs for ADHD and ASD. Children born to women with epilepsy had higher PRSs for ADHD (mean difference = 0.09, *P* = 0.07), not ASD (mean difference = 0.014, *P* = 0.78), compared to those without epilepsy. In these children, we observed stronger associations between the ADHD PRSs and neurodevelopmental traits closely linked to ADHD, specifically hyperactivity and inattention for all ages examined with the strongest association at 8 years. For the ASD PRSs, we observed stronger associations with motor development and language difficulties particularly at 5 years in children born to women with epilepsy. Although these associations were in the same direction and statistically significant for all ages in the general population, they did not reach statistical significance in children born to women with epilepsy. Findings from this study show that the ADHD and ASD PRSs were more strongly associated with hyperactivity/inattention, and with language/motor difficulties respectively in children born to women with epilepsy, compared to the general population. However, statistical significance was not reached likely due to limited sample size. These findings underscore the importance of considering genetic predisposition when assessing neurodevelopmental risks in this population.

## Teaser

Kinge-Rasmussen *et al.* found that associations between polygenic risk scores for attention-deficit/hyperactivity disorder and autism spectrum disorder and corresponding neurodevelopmental traits were stronger—but not statistically significant—among children of mothers with epilepsy compared with those in the general population.

## Graphical Abstract

**Graphical Abstract:**
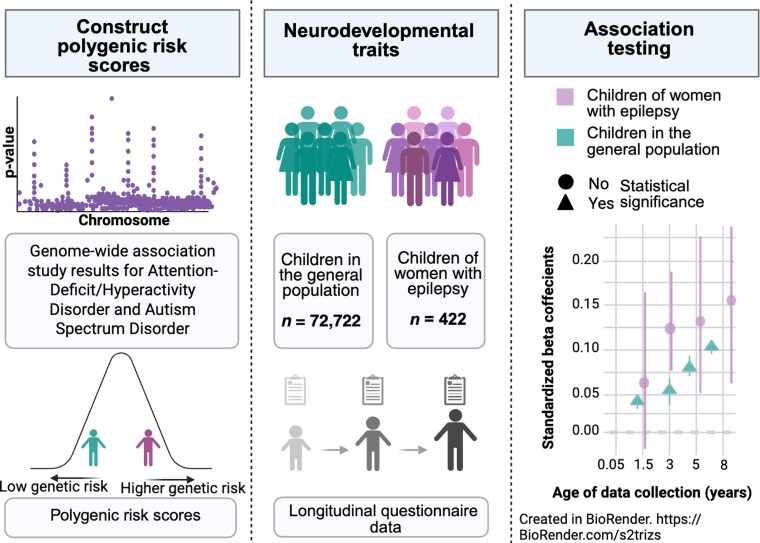

## Introduction

Globally, approximately 15 million women with epilepsy are of childbearing age, and most require treatment with antiseizure medications (ASMs) throughout pregnancy.^1^ Prenatal exposure to ASMs has been associated with congenital malformations and adverse neurodevelopmental outcomes such as attention-deficit/hyperactivity disorder (ADHD) and autism spectrum disorder (ASD) suggesting teratogenic effects.^2-5^ Seizures during pregnancy may be harmful, due to trauma from maternal falls, compromised blood supply, postictal hypoxia and lactic acidosis.^6^ This leaves the clinicians and pregnant women in a difficult dilemma, weighing the risk of seizure during pregnancy against the potential harmful long term effects associated with *in utero* ASM exposure.

ADHD and ASD are complex genetic disorders,^7,8^ with heritability estimates of 74–80% for ADHD^9^ and 64–91% for ASD.^10,11^ They are well known comorbidities in patients with epilepsy,^12-14^ as ADHD affects 22.3% of patients with epilepsy, while epilepsy occurs in 3.4% of individuals with ADHD.^15^ Similarly, the prevalence of ASD in patients with epilepsy is around 9.0%, while epilepsy affects 12.1% of patients with ASD.^16^ This comorbidity suggests underlying shared genetic risks,^17-20^ where associated genetic variants have been implicated in key neurobiological processes such as synaptic formation, remodelling and maintenance, neurotransmission, DNA methylation and chromatin remodelling.^17^

Genome-wide association studies (GWAS) identify single nucleotide polymorphisms (SNPs) associated with complex traits.^21,22^ Polygenic risk scores (PRSs) summarize an individual’s genetic susceptibility for a trait based on SNPs found in GWAS.^23^ PRSs have been widely implemented in child psychiatry research, and previous studies have identified associations between PRSs for ADHD and ASD with adverse neurodevelopmental traits in childhood within the general population.^24-26^ Understanding the genetic susceptibility for ADHD and ASD in children born to women with epilepsy is essential, as it may contribute to neurodevelopmental vulnerabilities independent of the risk associated with *in utero* ASMs exposure.

The objective of this study was to investigate whether PRSs for ADHD and ASD in children born to women with epilepsy differ from the general population and to explore if PRSs for ADHD and ASD are associated with neurodevelopmental traits from 0.5 years to 8 years in these children.

## Materials and methods

### Study population

This study uses data from the Norwegian Mother, Father and Child Cohort Study (MoBa) version 12 (2020). MoBa is an ongoing prospective, population-based pregnancy cohort with ∼114 000 children, 95 000 mothers and 75 000 fathers.^27^ Pregnant women in Norway between 1999 and 2008 were invited around Week 15 of the pregnancy.^28^ Blood samples were collected from the mothers, fathers and children (umbilical cord blood).^28^ Participants completed questionnaires during the pregnancy and childhood, and data were linked to the Medical Birth Registry of Norway (MBRN).

### Ethics statement

The study was approved by the Regional Committees for Medical and Health Research Ethics (2021/239237) and the Data Protection Officer at the Oslo University Hospital (2021/06870). All data were handled and in TSD (Tjenester for Sensitive Data), operated and developed by the IT Department, University of Oslo, Norway.

### Selection of samples

**Figure 1:**
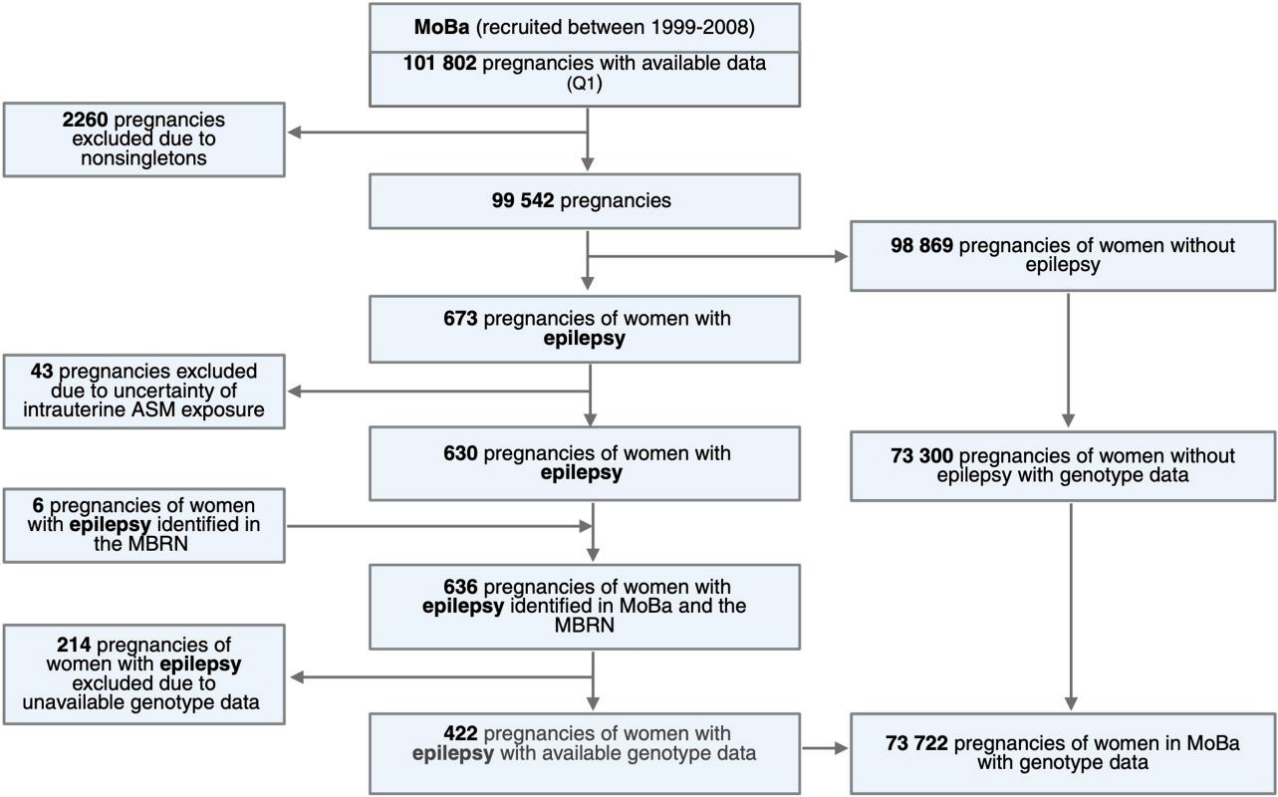
**Flow chart of sample selection.** MoBa, Mother, Father and Child Cohort Study; ASM, antiseizure medication; MBRN, Medical Birth Registry of Norway; Q1, MoBa questionnaire. Created in BioRender. Kinge-Rasmussen, M. (2025) https://BioRender.com/l19t961.

Children of women with epilepsy were identified using maternal responses to MoBa questionnaire Q1 (around Week 15 of gestation) and data from the Medical Birth Registry of Norway (MBRN). Only children from live and singleton births with were included (Fig. 1). By using Q1.39.47 regarding previous and current illnesses/health problems during pregnancy and associated medication use, we identified 673 children. *In utero* ASM exposure was defined by specific Anatomical Therapeutic Chemical (ATC) codes: N03A (ASMs), N05BA09 (clobazam) or S01EC01 (acetazolamide). Among the children of women with epilepsy, some were exposed to ASMs *in utero* (*n* = 277), while others were not (*n* = 145). Of the children exposed to ASMs *in utero*, the most used ASMs were carbamazepine (31%), lamotrigine (23%) and valproate (14%), and the majority of women (85%) used monotherapy. Forty-three children whose mothers reported epilepsy treatment without specifying the drug were excluded to avoid ASM exposure misclassification. Alternative modelling approaches, including multiple imputation, were considered. However, the missingness in the variable representing ASM exposure (ATC) code was considered to be missing not at random, indicating that imputation could introduce bias. An additional six children were identified from the MBRN increasing the total to 636 children, of whom 422 had available genotype data.

### Neurodevelopmental outcomes

The same outcome measures as described in Askeland *et al.*^26^ investigating the association between ADHD and ASD PRSs and neurodevelopmental traits in children of the general population of MoBa were chosen to enable replication in our larger cohort.

Data from the parent-reported questionnaires from pregnancy around Week 15 (Q1) and child ages 0.5 years (Q4), 1.5 years (Q5), 3 years (Q6), 5 years (Q5y) and 8 years (Q8y) were used. For descriptive statistics and details regarding on scales used, see Supplementary Table 1 and Supplementary Fig. 1. In brief, *social communication* items were assessed using the Ages and Stages Communication (ASQ)^29^ at 0.5 years. For *repetitive behaviour and social communication*, we used the Modified Checklist for Autism (M-CHAT)^30,31^ and Early Screening for Autistic Spectrum in Children (ESAT)^32^ at 1.5 years. At 3 and 8 years, the Social Communication Questionnaire (SCQ)^33^ was used, and at 5 years, the Childhood Asperger Syndrome Test (CAST)^34^ was used. *Motor difficulties* were measured using items from the ASQ. *Language difficulties* were measured using ASQ at 1.5, 3 and 5 years and Children Communication Checklist-2 (CCC-2)^35^ at 8 years. *Hyperactivity and inattention* were assessed using Child Behavior Checklist (CBCL)^36^ at 1.5 and 3 years; Conners Parent Rating Scale-Revised, Short Form (CPRS-R(s))^37^ at 5 years; and the Parent/Teacher Rating Scale for Disruptive Behavior Disorders (RS-DBD)^38^ at 8 years.

### Genotype data

Genotype data were available for a total of 73 722 children (50.9% were male) from singleton pregnancies. Pre-imputation, phasing, imputation and post-imputation quality control was performed by the PsychGen Center for Genetic Epidemiology and Mental Health at the Norwegian Institute of Public Health according to the published MoBaPsychGen pipeline v.1.^39^

### Statistical analysis

PRSs for ADHD and ASD were generated using *snp_ldpred2_auto()* from the *bigsnpR R* package.^40^ This PRS method uses a Bayesian approach that employs information from GWAS summary statistics and linkage disequilibrium (LD), to calculate risk effects of multiple genetic variants into a single predictive score.^40^ We used HapMap3+ variants of European individuals from the UK biobank as the reference LD panel.^41^ GWAS summary statistics were retrieved from European individuals in the Psychiatric Genomic Consortium (PGC) for ADHD (*n* = 38 691 cases, *n* = 186 843 controls)^42^ and ASD (*n* = 18 381cases, *n* = 27 969 controls).^43^ Quality control steps included removing SNPs with an INFO score ≤ 0.8 and minor allele frequency (MAF) ≤ 0.01 from the GWAS summary statistics.^23,40,44^ Additionally, SNPs with a misalignment in the standard deviations of the summary statics and the allele frequencies of the LD reference were removed, as these variants may invalidate an approximation of the LDpred-2.^44^ Except for varying the 50 values of *p* (polygenicity) evenly on a logarithmic scale between 0.0001 and 0.9, the default settings were used. As recommended by Prive *et al*.,^44^ the ‘allow_jump_sign’ option was deactivated to limit instability of the Gibbs sampler and ‘shrink_corr’ was set to 0.95. The resulting 50 chains were filtered to retain only chains providing the top imputed marginal scaled effect sizes, as defined by Privé *et al.*^41,44^; 45 out of 50 chains passed filtering for ASD, and all chains passed filtering for ADHD. Finally, the effect size estimates of the remaining chains were averaged before computing the PRSs. PRSs and all six trait measures were standardized to zero mean and a standard deviation of 1 prior to the analysis. *T.test()* function was used to perform a two-sample *t*-test to test the mean differences in PRSs for ADHD and ASD between children of mothers with epilepsy and children of mothers without epilepsy. Linear regressions using the *lm()* function were ran to test associations between PRSs for ADHD and ASD and the six neurodevelopmental traits. We included ASM exposure during the first trimester as a covariate to adjust for risk factors of the neurodevelopmental outcomes. To account for the gender difference with higher rates of ADHD and ASD diagnosis in males,^45^ sex was incorporated as a covariate in our analysis. To correct for multiple testing, false discovery rate (FDR) correction was applied using the *p.adjust*() function and the Benjamini–Hochberg method correcting for 25 tests per model run for each study group assessing the associations between the PRSs for ADHD and ASD and the neurodevelopmental outcome, with a significance threshold level of *P* < 0.05.^46^ All analyses were performed in *R* version 4.2.2.

## Results

### Neurodevelopmental characteristics

Table 1 provides the descriptive statistics for the neurodevelopmental scales. For details regarding min and max in each scale, see Supplementary Table 1.

**Table 1:** Descriptive statistics of the neurodevelopmental traits assessed

Age	Variable	*N* Children of mothers with epilepsy	*N* All children with available genotype data	Mean (SD) Children of mothers with epilepsy	Mean (SD) All children with available genotype data	No of items in scale
6 m	Social communication	350	62.588	5.52 (0.91)	5.44 (0.84)	5
	Motor development	351	62.597	6.95 (1.49)	6.79 (1.22)	6
18 m	Repetitive behaviour	278	49.689	6.51 (0.76)	6.35 (0.62)	6
	Social communication	278	49.661	15.52 (0.99)	15.45 (0.89)	15
	Language difficulties	299	53.371	4.49 (1.59)	4.21 (1.50)	3
	Motor development	299	53.436	6.90 (1.50)	6.71 (1.29)	6
	Hyperactivity	278	49.621	3.10 (1.00)	3.09 (0.97)	2
	Inattention	299	53.367	3.48 (1.03)	3.44 (0.96)	2
3 y	Repetitive behaviour	235	41.230	16.36 (2.72)	15.79 (2.49)	12
	Social communication	236	41.300	28.38 (2.03)	28.28 (1.78)	26
	Language difficulties	234	41.343	6.72 (1.25)	6.63 (1.11)	6
	Motor development	231	41.214	5.23 (1.37)	5.15 (1.32)	4
	Hyperactivity	234	41.228	6.22 (1.62)	5.23 (1.61)	4
	Inattention	234	41.245	3.16 (0.91)	3.17 (0.96)	2
5 y	Repetitive behaviour	65	10.800	5.55 (0.70)	5.41 (0.67)	5
	Social communication	65	10.808	11.89 (0.93)	11.69 (0.99)	11
	Language difficulties	157	28.956	6.66 (0.90)	6.68 (1.13)	6
	Motor development	157	28.940	13.10 (1.43)	12.97 (1.53)	12
	Hyperactivity	157	28.948	4.23 (1.48)	4.10 (1.31)	3
	Inattention	157	28.900	12.63 (3.98)	12.16 (3.42)	9
8 y	Repetitive behaviour	192	30.547	12.87 (1.47)	12.63 (1.12)	12
	Social communication	191	30.383	29.91 (2.63)	28.61 (2.44)	26
	Hyperactivity	191	30.516	12.92 (4.47)	12.46 (3.79)	9
	Inattention	191	30.523	14.24 (4.43)	13.85 (4)	9
	Language difficulties	192	30.443	22.56 (5.89)	21.22 (4.73)	16

### PRSs for ADHD and ASD associated with neurodevelopmental traits in the MoBa population

**Figure 2:**
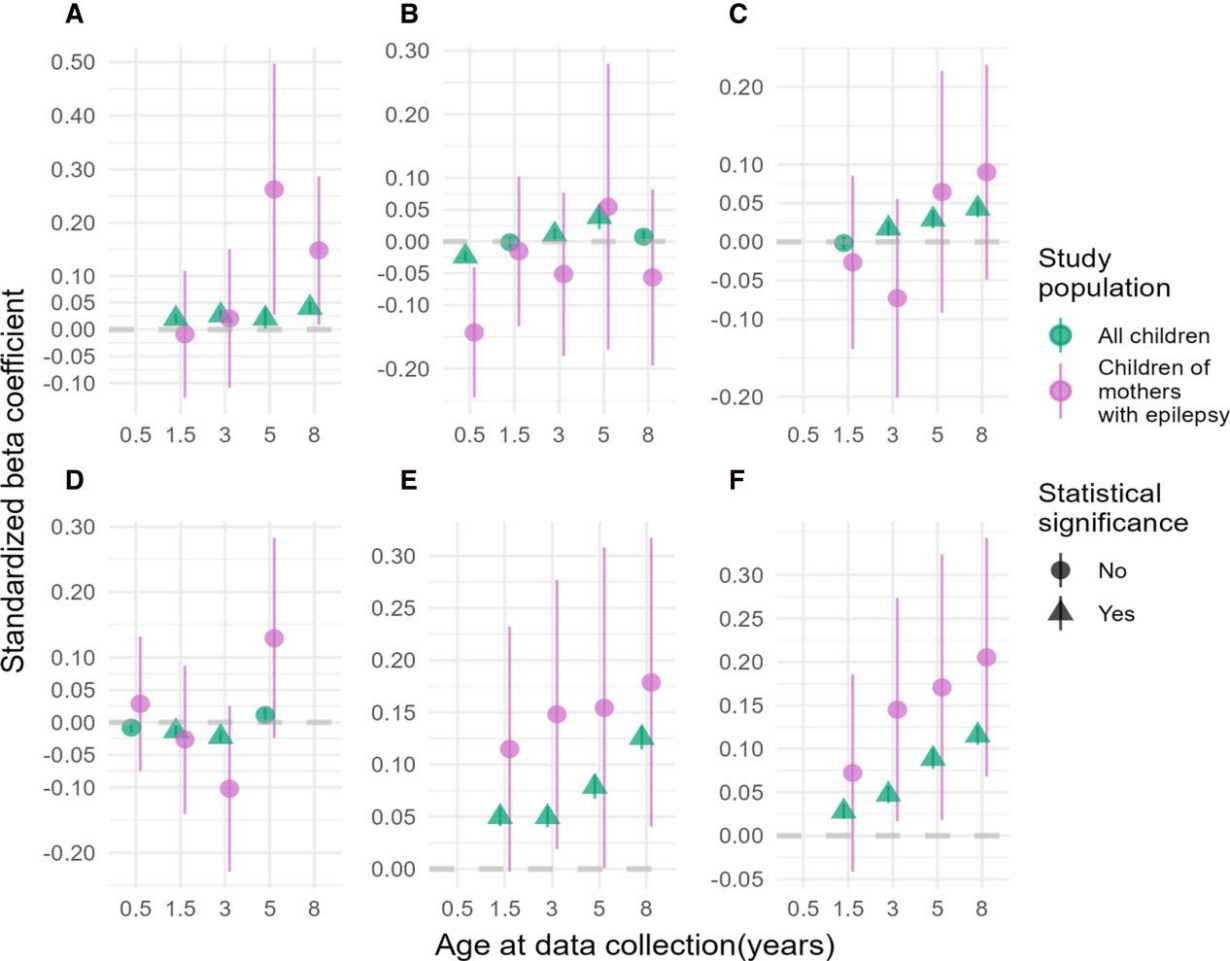
**PRSs for ADHD and associations with neurodevelopmental outcomes.** Plots of standardized *β* coefficients with 95% CIs bars from the linear regression models assessing the association between PRSs for ADHD and six neurodevelopmental traits in children of mothers with epilepsy (purple, *n* = 422) and all children with available genotype (green, *n* = 73 722). Significant associations (FDR < 5%) are visualized as triangles. (**A**) Repetitive behaviour, (**B**) social communication, (**C**) language difficulties, (**D**) motor difficulties, (**E**) hyperactivity and (**F**) inattention.

**Figure 3:**
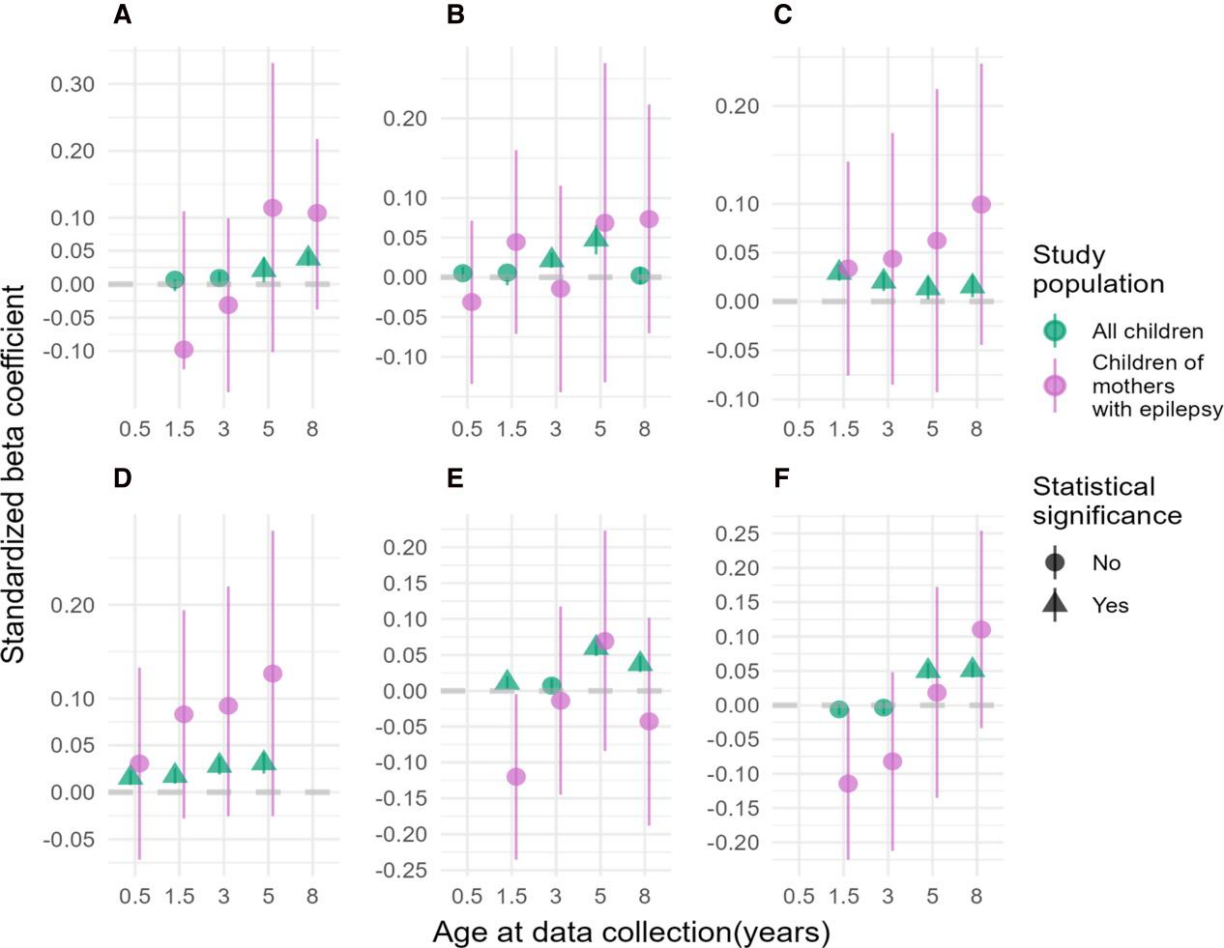
**PRSs for ASD and associations with neurodevelopmental outcomes.** Plots of standardized *β* coefficients with 95% CIs bars from the linear regression models assessing the association between six neurodevelopmental traits and PRSs for ASD in children of mothers with epilepsy (purple, *n* = 422) and all children with available genotype in MoBa (green, *n* = 73 722). Significant associations (FDR < 5%) are visualized as triangles. (**A**) Repetitive behaviour, (**B**) social communication, (**C**) language difficulties, (**D**) motor difficulties, (**E**) hyperactivity and (**F**) inattention.

When comparing our results with all children with available genotype data in MoBa (*n* = 73 722) to the previous study by Askeland *et al.*^26^ (*n* = 15 205), we successfully replicated the trends in associations between the PRSs for ADHD and ASD and all outcome measures, with far narrower 95% confidence intervals (CIs) (Figs 2A–F and 3A–F; Supplementary Tables 4 and 5).

### PRSs for ADHD associated with neurodevelopmental traits in children of women with epilepsy

**Figure 4:**
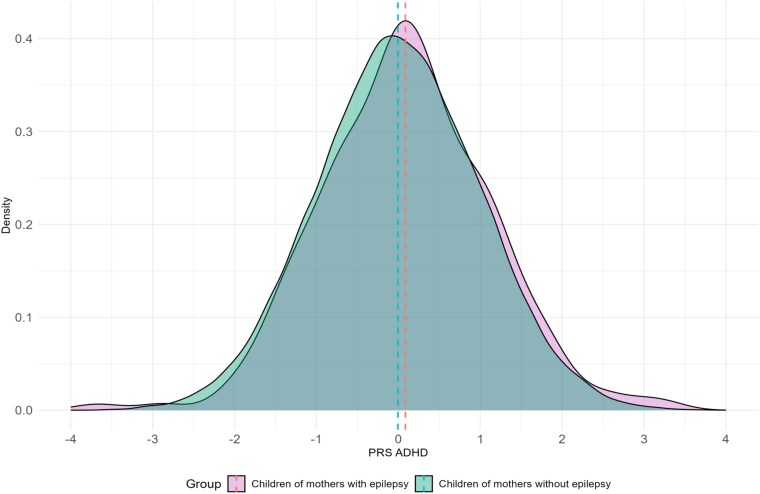
**PRSs for ADHD grouped by study population.** Density plots of standardized PRSs for ADHD in all children born to mothers with epilepsy (purple, *n* = 422) and children of mothers without epilepsy (green, *n* = 73 300). PRS, polygenic risk score; ADHD, attention-deficit/hyperactivity disorder.

The mean difference in ADHD PRSs between children of mothers with epilepsy (*n* = 422) and children of women without epilepsy (*n* = 73 300) was 0.09 (*P* = 0.07; Fig. 4). A right-skewed distribution indicated elevated ADHD PRSs in children of women with epilepsy.

The associations between ADHD PRSs and neurodevelopmental traits in children born to women with epilepsy and in children of the general population of MoBa are shown in Fig. 2A–F and Supplementary Tables 2 and 4. We detected stronger associations between ADHD PRSs and hyperactivity with increasing *β* coefficients as children aged, peaking at 8 years (*β* = 0.18, 95% CI = 0.04–0.31, FDR corrected *P* = 0.09) in children born of women with epilepsy compared to children of the general population (8 years *β* = 0.13, 95% CI = 0.11–0.14, FDR corrected *P* = 7.14E^−16^). A similar pattern was detected between ADHD PRSs and inattention in children of women with epilepsy (8 years *β* = 0.21, 95% CI = 0.07—0.34, FDR corrected *P* = 0.08) compared to the general population (8 years *β* = 0.12, 95% CI = 0.10–0.13, FDR corrected *P* = 7.14E^−16^). Notably, for the associations between ADHD PRSs and hyperactivity, and inattention at 3, 5 and 8 years and social communication 0.5 years and repetitive behaviour 5 years, the *P* values were <0.05 before FDR correction for multiple testing (Supplementary Table 2).

Despite consistent associations, adjusted *R*^2^ values indicated limited predictive power for neurodevelopmental traits in children of women with epilepsy, with most *R^2^* values falling below 6% (Supplementary Table 2). Notable exception was the PRSs for ADHD showed an adjusted *R*^2^ of 13% in predicting social communication at 5 years in children of women with epilepsy. This higher value should be interpreted with caution due to the small sample size of the subgroup (*n* = 65). Several models showed negative adjusted *R^2^* values, indicating that the models failed to explain the variability in the outcomes (Supplementary Table 2). Including ASM exposure as a covariate in the models generally did not increase the predictive power, with most changes in the adjusted *R*^2^ < 2%. The exception was ADHD PRSs associated with social communication at age 5 years, where the adjusted *R^2^* increased with 11% for these 65 children, 47 of whom were exposed to ASMs (Supplementary Table 2). Of note, a sensitivity analysis including the 43 children of women with epilepsy who were initially excluded due to uncertainty around *in utero* ASM exposure showed no significant changes in the associations (data not shown).

### PRSs for ASD associated with neurodevelopmental traits in children of women with epilepsy

**Figure 5:**
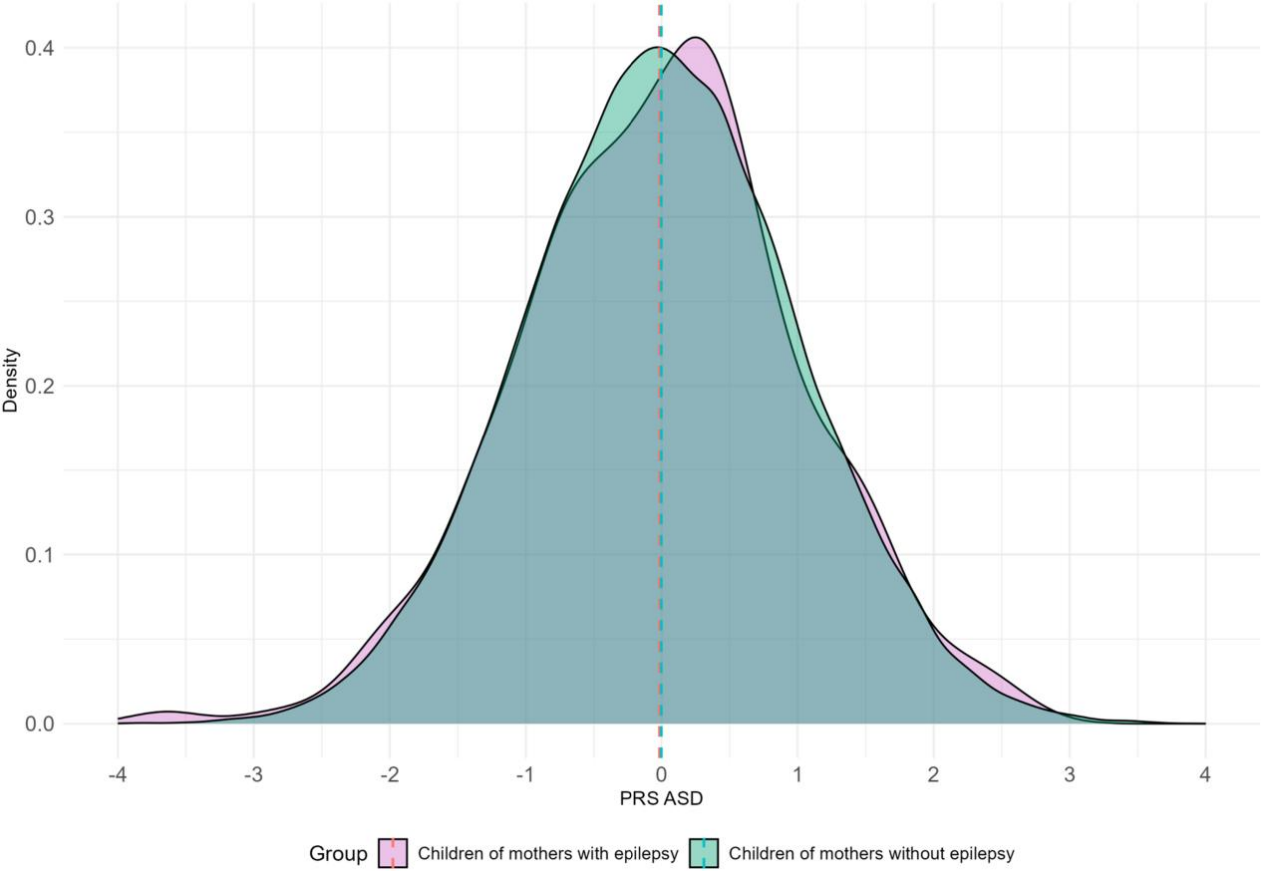
**PRSs for ASD grouped by study population.** Density plots of standardized PRSs for ASD in all children born to mothers with epilepsy (purple, *n* = 422) and children of mothers without epilepsy (green, *n* = 73 300). PRS, polygenic risk score; ASD, autism spectrum disorder.

We found a non-significant mean difference of 0.014 (*P* = 0.78) in ASD PRSs between in children of mothers with epilepsy compared to in children of mothers without epilepsy (Fig. 5**)**.

The associations between ASD PRSs and neurodevelopmental traits in children born to women with epilepsy and in children of the general population of MoBa are shown in Fig. 3A–F and Supplementary Tables 3 and 5. Children of mothers with epilepsy showed stronger associations between PRSs for ASD and language difficulties with consistently higher *β* coefficients increasing with the child age, with the strongest associations at 8 years (*β* = 0.10, 95% CI = −0.04–0.24, FDR corrected *P* = 0.48) compared to children of the general population (*β* = 0.02, 95% CI = 0.00–0.03, FDR corrected *P* = 1.15E^−2^). A similar trend was seen between PRSs for ASD and motor difficulties at 5 years in children of mothers with epilepsy (5 years *β* = 0.13, 95% CI = −0.02–0.28, FDR corrected *P* = 0.48) compared to children of the general population (5 years *β* = 0.03, 95% CI = 0.01–0.04, FDR corrected *P* = 1.23E^−7^). However, for the associations between motor difficulties at 0.5 and 1.5 and language difficulties at 5 years, we detected negative adjusted *R*^2^ values, indicating that the models do not explain the variability in the measured outcome traits (Supplementary Table 3). This was also detected in the associations between the ASD PRSs and social communication at 0.5 years and 3 years, repetitive behaviour at 3 and 5 years, hyperactivity at 3, 5 and 8 years and inattention at 5 years (Supplementary Table 3). Of note, the *P* values in the associations between the ASD PRSs and hyperactivity and inattention at 1.5 years were <0.05 before FDR correction (Supplementary Table 3).

The adjusted *R*^2^ values were all below 4%, indicating that the models had limited predictive power (Supplementary Table 3). We detected only small changes in adjusted *R*^2^ < 1% when including ASM exposure *in utero* as a covariate in the models, except for the association between the PRSs for ASD and social communication at 5 years where the adjusted *R^2^* increased to 13%. However, as mentioned, this group consists of only 65 individuals (Supplementary Table 3). A sensitivity analysis including 43 children of mothers with epilepsy initially excluded due to uncertainty around *in utero* ASM exposure showed no significant associations, confirming the stability of our initial findings (data not shown).

## Discussion

In this study, we examined the difference in the PRSs for ADHD and ASD in children of women with epilepsy compared to children from the general MoBa population and associations with six neurodevelopmental traits at 0.5–8 years. To our knowledge, this is the first study to assess polygenic risk for ADHD and ASD in children of mothers with epilepsy. These results provide important insights into genetic susceptibility to ADHD and ASD in these children, which is important for interpretation of pharmacoepidemiological studies on neurodevelopmental risks associated with *in utero* ASMs exposure.

### PRSs for ADHD and ASD in children of women with epilepsy

In general, we observed stronger associations between PRSs for both ADHD and ASD and all six neurodevelopmental traits at ages 5 and 8 years in the children of mothers with epilepsy compared to the general population. Notable exceptions included the association between ADHD PRSs and social communication at 8 years and ASD PRSs and inattention at 5 years and hyperactivity at 8 years. These findings indicate that the manifestations of genetic risks become progressively more pronounced as children become older, likely due to executive tasks becoming more challenging with age and/or greater precision in the assessment of the neurodevelopmental traits with age.^47^ Although formal clinical diagnoses were not assessed in this study, our findings align with the clinical observations, where the average age of diagnosis is 7 years for ADHD^48^ and 4 years and 4 months for ASD.^49^ Our results suggest that PRSs may be more predictive for the associated neurodevelopmental traits as children grow older.

Elevated ADHD PRSs in children born to mothers with epilepsy, coupled with stronger associations with hyperactivity and inattention, indicate a higher genetic susceptibility than in the general population. These findings enhance our understanding of the factors contributing to the increased ADHD risk in this population and suggest that for the neurodevelopmental traits related to ADHD the genetic risk may contribute more than previously recognized, beyond the direct effects of *in utero* ASM exposure.^50^ For ASD, PRSs showed stronger associations with language difficulties and motor difficulties as children age. However, negative adjusted *R^2^* values for motor development at 0.5 years and 1.5 years, and language difficulties at 5 years, indicate a lack of predictive ability in the models. Additionally, no difference in ASD PRSs in children of mothers with epilepsy versus children of mothers without epilepsy was detected. This is surprising given the well-known and high comorbidity of epilepsy and ASD, although lower than comorbidity of epilepsy and ADHD.^12,13^

The elevated PRSs observed in children born to women with epilepsy may also reflect shared heritable factors between epilepsy and neurodevelopmental disorders, as these conditions are thought to overlap in genetic architecture involving neuronal signalling and cortical development.^17-20^ Women with epilepsy may therefore carry a higher burden of common risk alleles related to ADHD and ASD, which may be transmitted to their offspring. In addition, gene-environment correlations could contribute, as maternal genetic liability may co-occur with epilepsy-related exposures during pregnancy, together amplifying neurodevelopmental risk. Moreover, factors such as socioeconomic disadvantages and comorbid psychiatric health conditions, which are known to be more prevalent among women with epilepsy,^51-53^ could further increase the risk of neurodevelopmental disorders in these children. This is particularly evident given that both parental psychiatric health conditions and poor socioeconomic status have been linked to increased risk of child neurodevelopmental disorders in the general population.^54-56^

### Intrauterine ASM exposure

Previous studies have shown associations between *in utero* exposure to ASMs, particularly valproate, topiramate and certain polytherapies with ASD, ADHD, intellectual disabilities (ID) and abnormal scores for gross motor skills and language impairment at 5 and 8 years.^2,4,57,58^ We did not identify an effect modification of ASM exposure on the associations between PRSs and neurodevelopmental. Several factors may explain this: ASM exposure may truly have no effect, the study may lack precision given the broad categorization of ASM used or the power may be insufficient due to the relatively small subset of children. The majority of the children were exposed to ASMs with low-risk profiles for neurodevelopmental outcomes, such as lamotrigine and moderate-risk such as carbamazepine, while a minority were exposed to higher-risk ASMs such as valproate, topiramate or polytherapies. PRSs for ADHD and ASD might influence neurodevelopmental traits independently of ASM exposure. However, specific types of ASMs, dosages or timing during pregnancy might still interact with genetic risks in ways not captured by our models. In addition, although previous studies have reported a dose-dependent increase in the risk of neurodevelopmental disorders following *in utero* exposures to ASMs such as valproate and topiramate,^4,59,60^ dosage could not be adjusted for, as this information is not available in the MoBa questionnaires. These considerations underscore the need for cautious interpretation of PRSs effects in complex trait studies and highlight the importance of accounting for potential environmental influences such as ASM exposures.

### Strengths and limitations

A major strength is the MoBa extensive longitudinal cohort study design, which includes genotyping data and comprehensive questionnaire-based assessments throughout childhood. Quantifying symptom load for each neurodevelopment trait allows for nuanced understanding beyond diagnostic categories. However, important limitations exist. GWAS capture common variants but miss rare variants with potential disease influence. Demographic and phenotype differences between the GWAS cohort and this cohort could affect the reliability of our PRSs. The MoBa’s longitudinal cohort study design is prone to study depletion,^61^ and the relatively small group of children born to women with epilepsy makes findings vulnerable to attrition and sampling bias. Limited number of participants receiving the 5-year questionnaire regarding social communication and repetitive behaviour impacts the models particularly among children of women with epilepsy. This study did not have information about clinical diagnosis of ASD or ADHD in the children or the women. Therefore, this information was not included in the analyses. Due to substantial overlap among the neurodevelopmental traits and assessing each association across multiple time points, we applied the Benjamini and Hochberg, FDR method for adjustment for multiple testing across all 25 tests. While this method reduces the risk of Type I errors, it may increase the probability of Type II errors, potentially masking genuine differences in the population due to overcorrection. These considerations are essential when interpreting the results.

### Future directions

Future studies should explore additional factors like other genetic variants, like rare variants, copy number variants (CNVs) and epigenetic modifications, to investigate genetic-environmental interactions. Additionally, examining associations using family-based designs (e.g. trios of mother, father and child) can deepen the understanding of heritability and transmission of risk factors.

## Conclusion

Findings from this study show that the ADHD and ASD PRSs were consistently more strongly associated with hyperactivity/inattention and with language/motor difficulties, respectively, in children born to women with epilepsy. However, statistical significance was not achieved, likely due to small sample size. These findings highlight the necessity of considering genetic predisposition in neurodevelopmental risk assessment. Nonetheless, since PRSs account for only a small portion of the variance, future research should incorporate genetic, epigenetic and environmental factors for improved risk prediction to develop early intervention and prevention strategies.

## Supplementary Material

fcag078_Supplementary_Data

## Data Availability

The code generated for the purpose of statistical analyses conducted in R is available at https://github.com/MathildeKinge-Rasmussen/Genetic-suceptibility-for-neurodevelopmental-disorders-in-children-born-to-women-with-epilepsy.git. The dataset originates from the Norwegian Mother, Father and Child Cohort study (MoBa), which is managed by the Norwegian Institutes of Public Health (NIPH). The data and materials required for scientific investigations or reproducibility can be made available to other researchers given an approval from the Regional Committees for Medical and Health Research Ethics in Norway, compliance with the General Data Protection Regulation and permission from NIPH under a data access agreement with MoBa. Applications can be submitted through helsedata.no.
