# Supplementary material for: Genetic susceptibility for neurodevelopmental disorders in children born to women with epilepsy
Source: Brain Commun. 2026 Mar 16;8(2):fcag078. doi: 10.1093/braincomms/fcag078 (PMC13022828; doi:10.1093/braincomms/fcag078)
Supplement: fcag078_Supplementary_Data [file fcag078_supplementary_data.pdf]

Supplementary material

Genetic susceptibility for neurodevelopmental disorders in children born to women with epilepsy

Table of contents

Supplementary Figure 1: Additional information about the neurodevelopmental scales used.......2
Supplementary Table 1: Descriptive statistics for measures of neurodevelopmental traits.......3
Supplementary Table 2: Table with results from the linear regressions investigating the associations between PRS for ADHD and the six neurodevelopmental traits for children of mothers with epilepsy.......4
Supplementary Table 3: Table with results from the linear regressions investigating the associations between PRS for ASD and the six neurodevelopmental traits for children of mothers with epilepsy.......5
Supplementary Table 4: Table with results from the linear regressions investigating the associations between PRS for ADHD and the six neurodevelopmental traits for all children from singleton pregnancies with available genotype data in MoBa.......6
Supplementary Table 5: Table with results from the linear regressions investigating the associations between PRS for ASD and the six neurodevelopmental traits for all children from singleton pregnancies with available genotype data in MoBa.......7

Supplementary Figure 1: Additional information about the neurodevelopmental scales

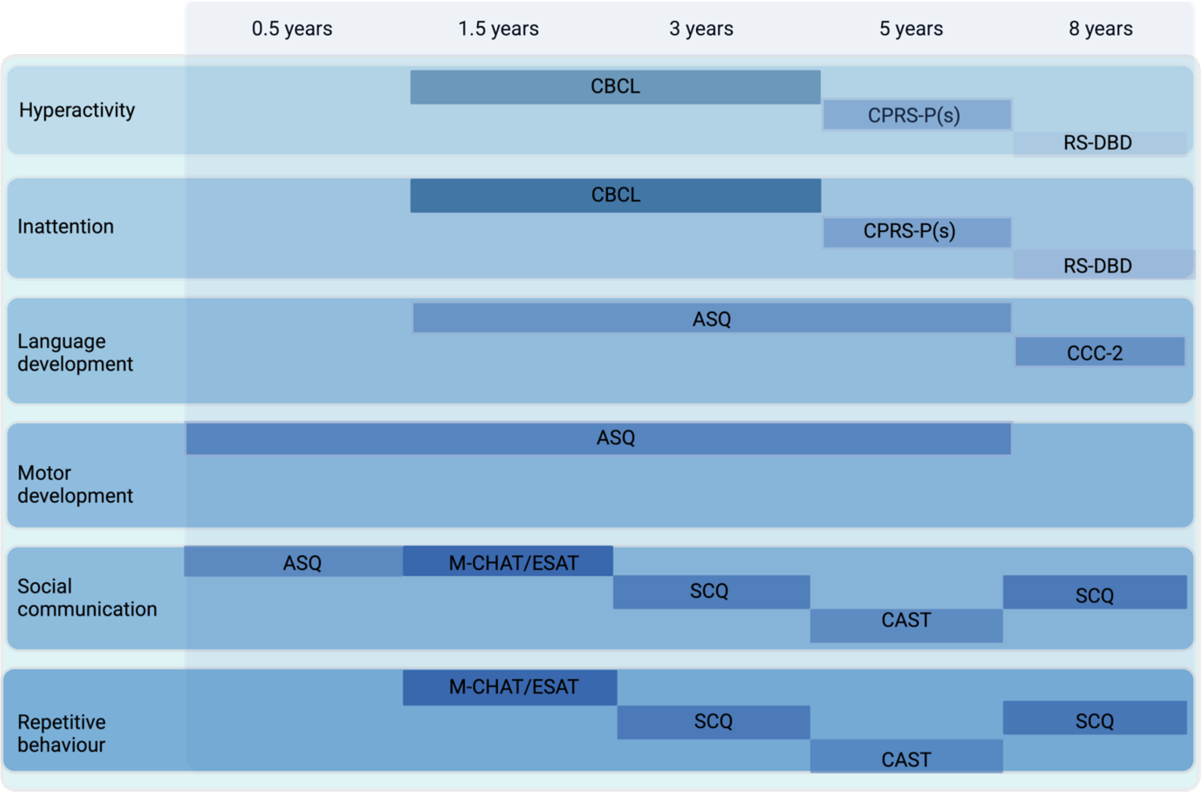

Figure 1: Overview of the neurodevelopmental scales used at the different ages to assess the six different neurodevelopmental traits. Abbreviations: ASQ: Ages and Stages Questionnaire; CAST: Childhood Asperger's Syndrome Test; CBCL: Child Behavior Checklist; CCC-2: Children's Communication Checklist-2; CDI: Child Developmental Inventory; CPRS-R (S): Conners Parent Rating Scale Revised, Short Form; ESAT: Early Screening of Autistic Traits Questionnaire; M-CHAT: Modified Checklist for Autism in Toddlers; RS-DBD: Parent/Teacher Rating Scale for Disruptive Behavior Disorders; SCQ: Social Communication Questionnaire. Created in BioRender. Kinge-Rasmussen, M. (2025) https://BioRender.com/0kr24kg.

Supplementary Table 1: Detailed descriptive statistics for measures of neurodevelopmental traits

Age	Variable	**N** All children* *(Children of mothers with epilepsy)*	**Mean** All children* *(Children of mothers with epilepsy)*	**SD** All children* *(Children of mothers with epilepsy)*	**Min** All children* *(Children of mothers with epilepsy)*	**Max** All children* *(Children of mothers with epilepsy)*	**No of items in scale**
6m	Social communication	62.588 (350)	5.44 (5.52)	0.84 (0.91)	5 (5)	20 (10)	5
	Motor development	62.597 (351)	6.79 (6.95)	1.22 (1.49)	6 (6)	24 (17)	6
18m	Repetitive behavior	49.689 (278)	6.35 (6.51)	0.62 (0.76)	6 (6)	11 (10)	6
	Social communication	49.661 (278)	15.45 (15.52)	0.89 (0.99)	15 (15)	30 (21)	15
	Language difficulties	53.371 (299)	4.21 (4.49)	1.50 (1.59)	3 (3)	9 (9)	3
	Motor development	53.436 (299)	6.71 (6.90)	1.29 (1.50)	6 (6)	18 (18)	6
	Hyperactivity	49.621 (278)	3.09 (3.10)	0.97 (1.00)	2 (2)	6 (6)	2
	Inattention	53.367 (299)	3.44 (3.48)	0.96 (1.03)	2 (2)	6 (6)	2
3y	Repetitive behavior	41.230 (235)	15.79 (16.36)	2.49 (2.72)	12 (12)	24 (24)	12
	Social communication	41.300 (236)	28.28 (28.38)	1.78 (2.03)	26 (26)	49 (45)	26
	Language difficulties	41.343 (234)	6.63 (6.72)	1.11 (1.25)	6 (6)	18 (18)	6
	Motor development	41.214 (231)	5.15 (5.23)	1.32 (1.37)	4 (4)	12 (10)	4
	Hyperactivity	41.228 (234)	5.23 (6.22)	1.61 (1.62)	4 (4)	12 (11)	4
	Inattention	41.245 (234)	3.17 (3.16)	0.96 (0.91)	2 (2)	6 (6)	2
5y	Repetitive behavior	10.800 (65)	5.41 (5.55)	0.67 (0.70)	5 (5)	10 (8)	5
	Social communication	10.808 (65)	11.69 (11.89)	0.99 (0.93)	11 (11)	21 (16)	11
	Language difficulties	28.956 (157)	6.68 (6.66)	1.13 (0.90)	6 (6)	16 (11)	6
	Motor development	28.940 (157)	12.97 (13.10)	1.53 (1.43)	12 (12)	24 (18)	12
	Hyperactivity	28.948 (157)	4.10 (4.23)	1.31 (1.48)	3 (3)	12 (12)	3
	Inattention	28.900 (157)	12.16 (12.63)	3.42 (3.98)	9 (9)	36 (36)	9
8y	Repetitive behavior	30.547 (192)	12.63 (12.87)	1.12 (1.47)	12 (12)	24 (22)	12
	Social communication	30.383 (191)	28.61 (29.91)	2.44 (2.63)	26 (26)	51 (41)	26
	Hyperactivity	30.516 (191)	12.46 (12.92)	3.79 (4.47)	9 (9)	36 (34)	9
	Inattention	30.523 (191)	13.85 (14.24)	4 (4.43)	9 (9)	36 (33)	9
	Language difficulties	30.443 (192)	21.22 (22.56)	4.73 (5.89)	16 (16)	62 (51)	16

Note: For each age, we calculated a mean score of the items corresponding to the specific neurodevelopmental traits, and participants with less than 50% missing items were included. For items where a low score reflected high symptom load, we reverse coded the items so that a high score reflected high symptom load for all traits. Note that data availability for the repetitive behavior and social communication variable at 5 years is only available for a subset of MoBa participants. This is because information regarding these psychometric instruments was only sent to a subset of MoBa participants. Abbreviations: *All children; all children from singleton pregnancies in MoBa with available genotype information.

Supplementary Table 2: Table with results from the linear regressions investigating the associations between PRS for ADHD and the six neurodevelopmental traits for children of mothers with epilepsy.

Age	Neurodevelopmental trait	N	Standardized *β*-coefficient	95% Confidence interval	*p*-value	FDR corrected *p*-value	Adjusted R^2 (*Before including ASM exposure as a covariate*)	Adjusted R^2(*After including ASM exposure as a covariate)*	Difference in adjusted R^2
6m	Social communication	350	-0,14	-0,25– (-0,04)	0,006 *	0,078	0,018	0,016	0.002
6m	Motor development	351	0,03	-0,074 – 0,13	0,583	0,736	-0,002	-0,001	-0.001
18m	Repetitive behavior	278	-0,01	-0,13–0,11	0,883	0,856	-0,007	-0,010	0,003
18m	Social communication	278	-0,02	-0.13–0.10	0,796	0,829	0,002	-0,001	0,003
18m	Language difficulties	299	-0,03	-0,14–0.09	0,639	0,736	0,0278	0,025	0,003
18m	Motor development	299	-0,03	-0,14–0.09	0,648	0,736	-0,005	-0,009	0,003
18m	Hyperactivity	278	0,11	-0,00–0.23	0,055´	0,139	0,008	0,005	0,003
18m	Inattention	299	0,07	-0.04–0.19	0,212	0,379	-0,000	-0,004	0,003
3y	Repetitive behavior	235	0,02	-0.11–0.15	0,755	0,821	-0,003	-0,006	0,003
3y	Social communication	236	-0,05	-0,18-0,08	0,430	0,597	0,004	0,000	0,004
3y	Language difficulties	234	-0,07	-0,20–0.06	0,264	0,441	0,028	0,024	0,004
3y	Motor development	231	-0,10	-0,23–0.03	0,116	0,241	0,046	0,046	0,000
3y	Hyperactivity	234	0,15	0,019–0.28	0,025*	0,103	0,013	0,001	0,003
3y	Inattention	234	0,15	0.02–0.27	0,027*	0,103	0,022	0,018	0,004
5y	Repetitive behavior	65	0,26	0,03–0,50	0,029*	0,103	0,068	0,053	0,015
5y	Social communication	65	0,05	-0.17–0.28	0,629	0,736	0,016	0,131	-0,115
5y	Language difficulties	157	0,06	-0.09–0.22	0,416	0,597	-0,007	-0,013	0,006
5y	Motor development	157	0,13	-0.02–0.28	0,098`	0,224	0,018	0,015	0,003
5y	Hyperactivity	157	0,15	0,00–0.31	0,049*	0,135	0,022	0,016	0,006
5y	Inattention	157	0,17	0,02–0.32	0,029*	0,103	0,023	0,025	-0,001
8y	Repetitive behavior	192	0,15	0,01–0.29	0,037*	0,116	0,014	0,008	0,005
8y	Social communication	191	-0,06	-0.20–0.08	0,421	0,597	0,021	0,017	0,004
8y	Hyperactivity	191	0,18	0.04–0.31	0,012*	0,097	0,029	0,028	0,001
8y	Inattention	191	0,21	0.07–0.34	0,004*	0,078	0,045	0,043	0.002
8y	Language difficulties	192	0,09	-0,05–0.23	0,203	0,379	0,012	0,008	0,005

Note: * Statistically significant finding

Supplementary Table 3: Table with results from the linear regressions investigating the associations between PRS for ASD and the six neurodevelopmental traits for children of mothers with epilepsy.

**Age**	**Neurodevelopmental trait**	**N**	**Standardized *β*-coefficient**	**95% Confidence interval**	***p*-value**	**FDR corrected *p*-value**	**Adjusted R^2 (*Before including ASM exposure as a covariate)***	**Adjusted R^2*(After including ASM exposure as a covariate)***	**Difference in adjusted R^2**
6m	Social communication	350	-0,03	-0,13–0,07	0,550	0,665	-0,005	-0,005	0,000
6m	Motor development	351	0,03	-0,07–0,13	0,558	0,665	-0,001	-0,004	0,002
18m	Repetitive behavior	278	-0,10	-0,13–0,11	0,098	0,484	0,003	0,000	0,003
18m	Social communication	278	0,04	-0,07–0,16	0,451	0,665	0,004	0,001	0,003
18m	Language difficulties	299	0,03	-0,08–0,14	0,545	0,665	0,028	0,025	0,003
18m	Motor development	299	0,08	-0,03–0,19	0,142	0,484	0,001	-0,002	0,003
18m	Hyperactivity	278	-0,12	-0,24– (-0.00)	0,042*	0,484	0,010	0,006	0,004
18m	Inattention	299	-0,11	-0,23– (-0.00)	0,043*	0,484	0,008	0,005	0,003
3y	Repetitive behavior	235	-0,03	-0,16–0,10	0,637	0,724	-0,003	-0,005	0,002
3y	Social communication	236	-0,01	-0,14–0,12	0,828	0,837	0,002	-0,002	0,004
3y	Language difficulties	234	0,04	-0,09–0,17	0,505	0,665	0,024	0,020	0,004
3y	Motor development	231	0,09	-0,04-0,22	0,155	0,484	0,041	0,044	-0,002
3y	Hyperactivity	234	-0,01	-0,15–0,12	0,837	0,837	-0,008	-0,012	0,004
3y	Inattention	234	-0,08	-0,21–0,05	0,215	0,538	0,006	0,003	0,003
5y	Repetitive behavior	65	0,11	-0,10–0,33	0,293	0,656	0,009	-0,006	0,015
5y	Social communication	65	0,07	-0,13–0,27	0,495	0,665	0,016	0,134	-0,118
5y	Language difficulties	157	0,06	-0,09–0,22	0,428	0,665	-0,008	-0,013	0,005
5y	Motor development	157	0,13	-0,06–0,28	0,102	0,484	0,016	0,015	0,001
5y	Hyperactivity	157	0,07	-0,08–0,22	0,374	0,665	0,001	-0,004	0,006
5y	Inattention	157	0,02	-0,14–0.17	0,813	0,837	-0,012	-0,006	-0,006
8y	Repetitive behavior	192	0,11	-0,04–0,25	0,146	0,484	0,002	-0,004	0,005
8y	Social communication	191	0,07	-0,07–0,22	0,316	0,656	0,021	0,019	0,002
8y	Hyperactivity	191	-0,04	-0.18- 0.10	0,559	0,665	-0,008	-0,004	-0,004
8y	Inattention	191	0,11	-0,03-0,25	0,133	0,484	0,014	0,010	0,004
8y	Language difficulties	192	0,10	-0,04-0,24	0,175	0,486	0,014	0,009	0,005

Note:* Statistically significant finding

Supplementary Table 4: Table with results from the linear regressions investigating the associations between PRS for ADHD and the six neurodevelopmental traits for all children with available genotype data in MoBa.

Age	Neurodevelopmental trait	N	Standardized *β*-coefficient	95% Confidence interval	*p*-value	FDR corrected *p*-value	Adjusted R^2
6m	Social communication	62,588	-0,02	-0,032— (-0,02)	3,81e-09*	8,65e-09*	0,001
6m	Motor development	62,597	-0,01	-0.02– (-0.00)	0,0424	5,30e-02	0,000
18m	Repetitive behavior	49,689	0,02	0,01–0,03	7,34e-06*	1.22e-05*	0,001
18m	Social communication	49,661	0,00	-0.01–0.01	0,766	7,66e-01	0,002
18m	Language difficulties	53,371	0,00	-0.01–0.01	0,691	7.19e-01	0,038
18m	Motor development	53,436	-0,01	-0,02– (-0,01)	0,00167*	2,31e-03*	0,001
18m	Hyperactivity	49,621	0,05	0,04–0.06	<2e-16*	7,14e-16*	0,005
18m	Inattention	53,367	0,03	0,02–0,04	1,33e-10*	3.25e-10*	0,003
3y	Repetitive behavior	41,230	0,03	0,02–0,04	7,64e-08*	1.59e-07*	0,010
3y	Social communication	41,300	0,01	0,00—0.02	0,0239*	3,14e-02*	0,011
3y	Language difficulties	41,343	0,02	0,01-0.03	0,000332*	4,88e-04*	0,012
3y	Motor development	41,214	-0,02	-0,03– (-0.01)	2,32e-06*	4.14e-06*	0,069
3y	Hyperactivity	41,228	0,05	0,04– 0.06	<2e-16*	7,14e-16*	0,003
3y	Inattention	41,245	0,05	0,04–0.06	<2e-16*	7,14e-16*	0,003
5y	Repetitive behavior	10,800	0,02	0.00–0.04	0,0344*	4,52e-02*	0,005
5y	Social communication	10,808	0,04	0.02–0.06	6,23e-05*	9,73e-05*	0,006
5y	Language difficulties	28,956	0,03	0.02–0.04	7,2e-07*	1,38e-06*	0,007
5y	Motor development	28,940	0,01	0,00–0.02	0,0452*	5,380952e-02	0,077
5y	Hyperactivity	28,948	0,08	0,07–0.09	<2e-16*	7,14e-16*	0,016
5y	Inattention	28,900	0,09	0,08–0.10	<2e-16*	7,14e-16*	0,022
8y	Repetitive behavior	30,547	0,04	0,03–0.05	1,25e-12*	3.47e-12*	0,007
8y	Social communication	30,383	0,01	-0,00–0.02	0,185	2.10e-01	0,015
8y	Hyperactivity	30,516	0,13	0,11–0.14	<2e-16*	7,14e-16*	0,034
8y	Inattention	30,523	0,12	0,10–0.13	<2e-16*	7,14e-16*	0,042
8y	Language difficulties	30,443	0,04	0,03–0.05	3,84e-14*	1.2e-13*	0,011

Note:* Statistically significant finding

Supplementary Table 5: Table with results from the linear regressions investigating the associations between PRS for ASD and the six neurodevelopmental traits for all children with available genotype data in MoBa.

Age	Neurodevelopmental trait	N	Standardized *β*-coefficient	95% Confidence interval	*p*-value	FDR corrected *p*-value	Adjusted R^2
6m	Social communication	62,588	0,01	-0,00–-0,01	0,197	2,14e-01	0,001
6m	Motor development	62,597	0,02	0,01–0,02	9,7e-05*	1.86e-04*	0,000
18m	Repetitive behavior	49,689	0,01	-0,01–0,01	0,130	1,71e-01	0,000
18m	Social communication	49,661	0,01	-0,01–0,01	0,19	2,14e-01	0,002
18m	Language difficulties	53,371	0,03	0,02–0,04	3,77e-12*	2,35e-11*	0,039
18m	Motor development	53,436	0,02	0,001–0.03	4,69e-05*	9,7e-05*	0,001
18m	Hyperactivity	49,621	0,01	0,00–0,02	0,0104*	1,73e-02*	0,003
18m	Inattention	53,367	-0,01	-0,01–0,00	0,147	1,75e-01	0,001
3y	Repetitive behavior	41,230	0,01	-0,00–0.02	0,0575	7,8e-02	0,009
3y	Social communication	41,300	0,02	0,01–0.03	1,14e-05*	2,85e-05*	0,011
3y	Language difficulties	41,343	0,02	0,01–0,03	3,09e-05*	7,02e-05*	0,012
3y	Motor development	41,214	0,03	0,02–0.04	2,59e-09*	9,25e-09*	0,069
3y	Hyperactivity	41,228	0,01	-0,00–0,02	0,1402	1,70e-01	0,000
3y	Inattention	41,245	0,00	-0,01–0,01	0,456850	4,75e-01	0,000
5y	Repetitive behavior	10,800	0,02	0,00–0,04	0,0262*	3,85e-02*	0,005
5y	Social communication	10,808	0,05	0,03–0,07	6,74e-07*	1,87e-06*	0,007
5y	Language difficulties	28,956	0,01	0,00–0,02	0,021*	3,28e-02*	0,006
5y	Motor development	28,940	0,03	0,02–0,04	3,94e-08*	1,23e-07*	0,078
5y	Hyperactivity	28,948	0,06	0,05–0.07	<2e-16*	1,67e-15*	0,014
5y	Inattention	28,900	0,05	0,04–0,06	<2e-16*	1,66e-15*	0,017
8y	Repetitive behavior	30,547	0,04	0,02–0.05	1,41e-11*	7,05e-11*	0,007
8y	Social communication	30,383	0,00	-0,01–0,01	0,718	7.18e-01	0,015
8y	Hyperactivity	30,516	0,04	0,03–0,05	4,26e-11*	1.77e-10*	0,019
8y	Inattention	30,523	0,05	0,04–0,06	<2e-16*	1,66e-15*	0,031
8y	Language difficulties	30,443	0,02	0,00–0,03	0,00648*	1,15e-02*	0,010

Note:* Statistically significant finding
